# Risk factors associated with suicide in adolescents and young adults (AYA) with cancer

**DOI:** 10.1002/cam4.4246

**Published:** 2021-09-29

**Authors:** Sarah Heynemann, Kate Thompson, Donovan Moncur, Sandun Silva, Madawa Jayawardana, Jeremy Lewin

**Affiliations:** ^1^ Department of Medical Oncology Peter MacCallum Cancer Centre Melbourne Victoria Australia; ^2^ ONTrac at Peter Mac Victorian Adolescent and Young Adult Cancer Service Peter MacCallum Cancer Centre Melbourne Victoria Australia; ^3^ Department of Psychiatry Peter MacCallum Cancer Centre Melbourne Victoria Australia; ^4^ Department of Statistics, Data Science and Epidemiology Swinburne University of Technology Hawthorn Victoria Australia; ^5^ NHMRC Clinical Trials Centre University of Sydney Sydney New South Wales Australia; ^6^ Office of Cancer Research Peter MacCallum Cancer Centre Melbourne Victoria Australia; ^7^ Sir Peter MacCallum Department of Oncology The University of Melbourne Parkville Victoria Australia

**Keywords:** adolescents and young adults, cancer, suicidality, suicide

## Abstract

**Background:**

Higher rates of death by suicide are recognized both in individuals of any age with cancer and, separately, among adolescents and young adults (AYA) without cancer. Given this intersection, identifying risk factors associated with suicidal risk among AYA with cancer is critical.

**Objective:**

To identify characteristics associated with suicide among AYA with cancer.

**Methods:**

A retrospective analysis of AYA (aged 15–39) during 1975–2016 from the Surveillance, Epidemiology, and End Results database was conducted. Clinical and demographic factors associated with death by suicide among the AYA cancer population were compared to (i) US population normative data (standardized mortality ratios [SMRs]) and (ii) other AYA individuals with cancer (odds ratios).

**Results:**

In total, 922 suicides were found in 500,366 AYA with cancer (0.18%), observed for 3,198,261 person‐years. The SMR for AYA with cancer was 34.1 (95% confidence interval [CI]: 31.4–36.9). Suicide risk was particularly high in females (SMR = 43.4, 95% CI: 37.2–50.4), unmarried persons (SMR = 50.6, 95% CI: 44.7–57.1), those with metastatic disease (SMR = 45.2, 95% CI: 33.1–60.3), or certain histological subtypes (leukemia, central nervous system, and soft tissue sarcoma). Risk generally reduced over time, however remained elevated ≥5 years following a cancer diagnosis (SMR > 5 years = 28.1, 95% CI: 25.4–31.0). When comparing those who died from suicide and those who did not, the following factors demonstrated significant associations: sex (males > females), race (White ethnicity > Black/other ethnicity), relationship status (never married > other), and disease stage (distant > localized).

**Conclusions:**

Death due to suicide/non‐accidental injury is high compared to normative data, requiring increased awareness among health‐care providers, suicide risk monitoring in AYA, and appropriately tailored psychosocial interventions.

## Graphical abstract

Individuals diagnosed with cancer whilst adolescents or young adults have a more than 30 times increased risk of death by suicide/non‐accidental injury than the general population. This risk remains elevated ≥5 years following a cancer diagnosis necessitating awareness among both oncology and primary care health‐care providers.

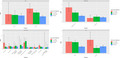

## 1 INTRODUCTION

Adolescents and young adults (AYA) with cancer are a unique population in that they represent a cohort with specific biological and tumor characteristics, occurring during a period of significant psychosocial and neurodevelopmental transition.^1^ Approximately 70,000 AYA (a demographic conventionally defined as age 15–39 years) are diagnosed with a malignancy each year within the United States (US),^2^ and cancer represents the greatest cause of disease‐related death within this population.^3^ Within the AYA population, only suicide, homicide, and unintentional injury claim more lives per year.^4^ With survival rates now approximating 85% for most major tumor types,^2^, ^5^ increased focus on the survivorship needs of this population is required, including the non‐malignant causes of morbidity and mortality.

Individuals with a current or prior diagnosis of cancer have an increased risk of death by suicide compared to the general population.^6^, ^7^ A retrospective US population‐based study, reported a rate of suicide of 28.9/100,000 person‐years in the over 8.6 million patients diagnosed with cancer during 1973–2014, with a standardized mortality ratio (SMR) of 4.4.^7^ In addition, patients diagnosed with cancer <40 years were over 30 times more likely to have died by suicide than the general population.^7^

Receiving a cancer diagnosis at any age may prove confronting for a range of reasons including the disruption this represents to a person’s expected life trajectory. For AYA, a cancer diagnosis and its management may pose a variety of unique concerns. These include disruptions to educational and vocational goals, financial burdens, changes in emotional well‐being, treatment impacts on fertility and sexuality, physical changes or body image concerns, relationship adjustments, and confrontation of the possibility of very premature mortality.^1^, ^8^

Higher rates of death by suicide are recognized both in individuals of any age with cancer and, separately, in AYA without cancer.^9^ Considerably elevated rates of death by suicide among AYA with cancer should therefore come as no surprise, and this has previously been reported.^7^ There is, however, a pressing need to identify and further evaluate factors which may place AYA at particular risk.^10^, ^11^ This study, therefore, sought to investigate clinical and demographic factors which were associated with death by suicide among the AYA cancer population compared to: (i) US population normative data and (ii) other AYA individuals with cancer.

## 2 METHODS

### 2.1 Study population

**FIGURE 1:**
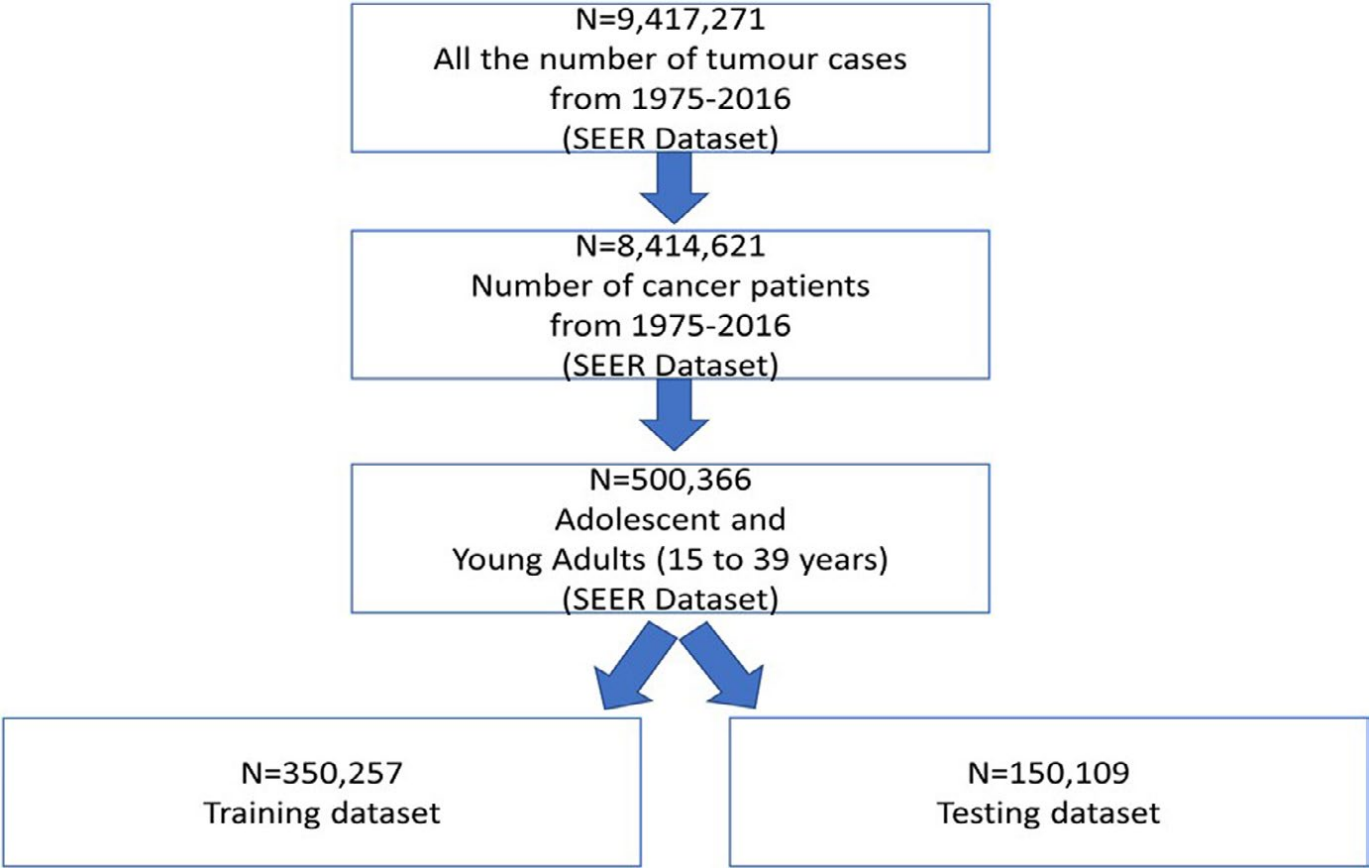
Consort diagram SEER dataset. SEER, Surveillance, Epidemiology, and End Results

This study was performed using data from the Surveillance, Epidemiology, and End Results (SEER) program.^12^ This program collects cancer incidence data from population‐based cancer registries encompassing approximately 35% of the entire US population.^13^ SEER releases data submissions annually, containing new incidence data and updated information for existing cases. Our study is based on the November 2018 submission with data from 1975 to 2016 (*N* = 9,417,271 tumor cases, Figure 1). Comparisons with the general US population were based on mortality data collected for all causes of death by the National Center for Health Statistics,^14^ a dataset spanning from 1975 to 2016 which is accessible using the SEER*Stat program.

### 2.2 Dataset

Surveillance, Epidemiology, and End Results includes different registries covering various time periods and demographical areas. SEER 9 database of the 2018 submission includes nine registries covering the time period 1975–2016. This database was used to calculate SMRs cross‐sectionally and longitudinally (0–1 years post‐cancer diagnosis, 1–5 years, >5 years). For cross‐sectional analysis, data are presented for categories in which 100,000 person‐years or more of survival time have been accrued, as has been reported previously.^6^, ^7^

Surveillance, Epidemiology, and End Results 18 database of the 2018 submission includes 18 registries encompassing cases from 2000 to 2016. This includes 500,366 patients aged between 15 and 39 years, 922 (0.18%) of whom died due to suicide/self‐inflicted injury. This dataset was used to calculate odds ratios (ORs).

The SEER dataset includes sex, age at diagnosis, year of diagnosis, number of primary tumors, interventions received (i.e., surgery, radiotherapy, and chemotherapy), cancer site, and tumor grade variables. However, data on radiotherapy, chemotherapy interventions, comorbid physical or mental health conditions, and social demographics were not available for SMR calculations.

Patients were considered to have died by suicide if the cause of death variable was coded as “Suicide and Self‐inflicted Injury (50220),” while patients with other causes of death, or who were alive at follow‐up, were not classified as deaths due to suicide. Thus, a dichotomous outcome variable was constructed (0 = alive/death due to other or unknown causes and 1 = death due to suicide or self‐inflicted injury).

### 2.3 Statistical analysis

In order to identify demographic and clinical factors associated with death by suicide among AYA cancer patients; SMRs and ORs were calculated. SMR is defined as the number of cause specific or total deaths in a given population as a percentage of the deaths that would have been expected if the age‐ and sex‐specific rates in a “standard” population were applied.^15^ SMRs were calculated for specific demographic and clinical factors. The effects of demographic and clinical factors were additionally analyzed in relation to death by suicide in the AYA group via ORs using the SEER 18 database.

Surveillance Research Program, National Cancer Institute SEER*Stat software (seer.cancer.gov/seerstat) version 8.3.6.1 was used to extract data related to AYA with cancer and to calculate SMRs. All statistical analyses were performed using R statistical software (Vienna, Austria), version 3.6.1.^16^ Two‐sided *p* values of <0.05 were considered statistically significant.

## 3 RESULTS

A total of 922 deaths (0.18%) from suicide/non‐accidental injury were found in 500,366 AYAs with cancer observed for 3,198,261 person‐years. Overall, AYA with cancer were 34 times more likely than the general population to have died by suicide/self‐inflicted injury (SMR 34.1: 95% CI 31.4–36.9; Table 1). Elevated SMRs were identified across all collected socio‐demographic and tumor variables, although were particularly high in: females (SMR 43.5: 95% CI 37.2–50.4); single relationship status (SMR 50.6, 95% CI 44.7–57.1), those with metastatic disease (SMR 45.2, 95% CI 33.1–60.3), or for those with particular tumor types (leukemia [SMR 65.3, 95% CI 38.7–103.1], central nervous system [CNS] and associated tumors [SMR 67.6, 95% CI 45.3–97.1], or soft tissue sarcoma [SMR 79.3, 95% CI 60.8–101.7]).

**TABLE 1:** Incidence of deaths by suicide/self‐inflicted injury in AYA with cancer by demographic and clinical characteristics

Variable	Value	Total^a^	Suicide^a^	PY^b^	SMR^b^	95% CI^b^
All age groups		8,414,621	13,278	26,540,984.64	4.52	(4.42–4.63)
All AYA patients with cancer	All patients	500,366	922	3,198,261	34.08	(31.41–36.91)
Sex	Male	204,963	642	1,220,562	31.36	(28.47–34.48)
	Female	295,403	280	1,977,699	43.38	(37.16–50.35)
Race	White	398,215	844	2,728,845	34.4	(31.61–37.37)
	Black	51,407	32	253,022	18.17	(11.25–27.77)
	American Indian/Alaskan Native/Asian or Pacific Islander/ Unknown	50,744	46	216,394	98.45	(60.14–152.05)
Relationship status	Single (never married)	205,086	410	1,079,965	50.64	(44.7–57.14)
	Married	228,266	358	1,690,555	24.48	(21.48–27.78)
	Separated/divorced/widowed/unmarried	31,071	77	219,636	31.92	(23.69–42.08)
	Unknown	35,943	77	208,105	44.41	(32.86–58.72)
Age at diagnosis	15–19	32,313	46	215,921.17	46.16	(32.15–64.2)
	20–24	52,727	110	368,433.00	51	(39.94–63.06)
	25–29	86,160	161	604,311.95	39	(31.88–46.84)
	30–34	132,669	247	854,155.07	36	(30.66–41.66)
	35–39	196,497	358	1,155,439.44	27	(23.36–30.76)
Year of diagnosis	1975–1984	47,140	178	978,407.29	19	(16.35–22.15)
	1985–1994	75,225	282	1,046,850.79	39	(33.7–43.96)
	1995–2004	140,448	279	789,584.20	56	(46.74–66.25)
	2005–2016	237,553	183	383,418.34	177	(138.62–223.12)
Number of primary tumors	Single	451,716	855	2,679,835	35.9	(33.01–38.96)
	Multiple	48,650	67	518,426	17.33	(11.69–24.74)
Surgery	Unknown	7331	23	50,378	44.73	(17.98–92.17)
	Not performed	125,111	258	514,137	46.05	(39.48–53.4)
	Surgery performed	367,924	641	2,633,745	30.64	(17.98–33.71)
Cancer site	Leukemias	22,797	26	74,038	65.3	(38.7–103.13)
	Lymphomas	59,763	131	407,365	36.23	(29.38–44.2)
	CNS/intracranial/intraspinal neoplasms	21,745	46	101,859	67.6	(45.28–97.09)
	Osseous and chondromatous neoplasms	7053	16	38,636	25.77	(13.32–45.01)
	Soft tissue sarcomas	29,410	93	143,412	79.29	(60.79–101.65)
	Germ cell and trophoblastic neoplasms	44,298	142	357,489	24.99	(20.24–30.52)
	Melanoma and skin carcinomas	53,448	107	456,355	26.17	(20.36–33.12)
	Carcinomas	252,386	343	1,574,845	33.03	(28.74–37.77)
	Miscellaneous specified neoplasms, NOS	6644	10	32,191.08	48.96	(13.34–125.35)
	Unspecified malignant neoplasms/ unclassified	2822	8	12,070	70.93	(8.59–256.24)
Grade	Well differentiated; Grade I	37,808	61	252,486	24.17	(17.35–32.8)
	Moderately differentiated; Grade II	63,142	94	335,472	36.22	(27.57–46.72)
	Poorly differentiated; Grade III	63,868	81	278,417	29.93	(21.75–40.18)
	Undifferentiated anaplastic; Grade IV	17,479	27	74,335	48.28	(27.6–78.41)
	Other cell (T‐, B‐, Null, N K)	31,073	43	90,812.76	53.94	(34.19–80.93)
	Unknown	286,996	616	2,166,737.12	34.59	(31.36–38.07)
SEER Historic Stage A (1973–2015)	Localized	203,648	392	1,731,734.98	29.1	(25.68–32.85)
	Regional	96,577	148	588,660.98	28.1	(22.54–34.63)
	Distant	63,066	75	190,979.02	45.2	(33.1–60.3)
	Unstaged/ unknown	137,075	75	686,885.64	45.63	(39.61–52.3)

Abbreviations: AYA, adolescents and young adult; CNS, central nervous system; NOS, not otherwise specified; PY, person‐years; SEER, Surveillance, Epidemiology, and End Results; SMR, standardized mortality ratio.

^a^ Incidence––SEER 18 Regs Research Data + Hurricane Katrina Impacted Louisiana Cases, Nov 2018 Sub (1975–2016 varying).

^b^ SEER 9 Regs Research Data, Nov 2018 Sub (1975–2016) for SMRs.

**FIGURE 2:**
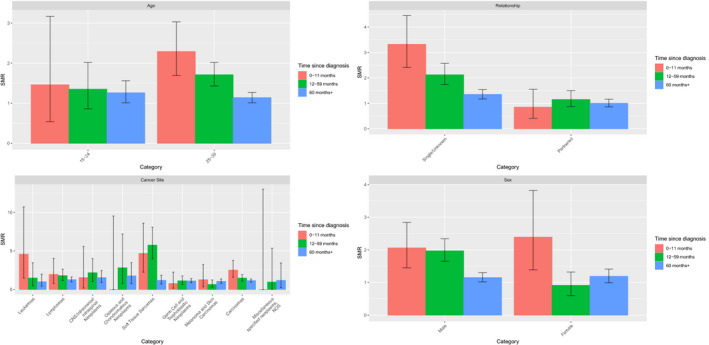
Incidence of death by suicide/self‐inflicted injury in AYA with cancer by demographic and clinical characteristics considered longitudinally from the date of cancer diagnosis (NB: Categories where SMR = 0 which do not have patients recorded for the specific time category have been omitted. See Data S1 for further detail). AYA, adolescents and young adult; SMR, standardized mortality ratio

Longitudinal SMR analysis was performed by grouping data into three time periods calculated from the date of cancer diagnosis (0–1 year, 1–5 years, >5 years) (Figure 2; Data S1). Overall, in the first 12 months following a cancer diagnosis, AYA were over 70 times (SMR 72.8; 95% CI 54.7–95.0) more likely to die by suicide/self‐inflicted injury with risk decreasing over time. The risk of death by suicide/non‐accidental injury remained above background population risk even beyond 5 years with an SMR of 28.1 (95% CI 25.4–31.0). Reduction of risk with duration of time post‐treatment was identified in most socio‐demographic and tumor variables studied with a few exceptions of note. For example, for patients diagnosed with cancer while 15–19 years old, risk of death by suicide was contrastingly highest 5 years after their cancer diagnosis (SMR 49.9: 95% CI 33.4–71.7).

**FIGURE 3:**
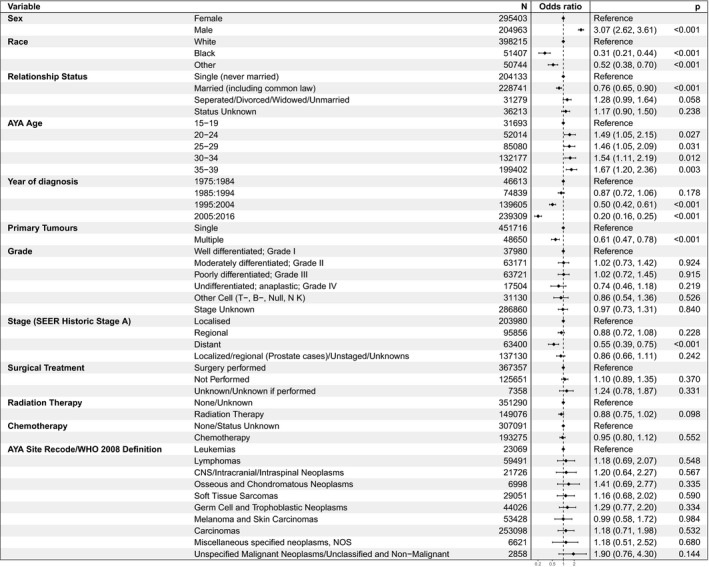
Factors affecting deaths due to suicide/self‐inflicted injury in AYA patients with cancer by demographic and clinical characteristics. AYA, adolescents and young adult

A comparison was conducted between AYA cancer patients who had died due to suicide/self‐inflicted injury and other AYA cancer patients (Figure 3). Regarding demographic characteristics, there was an association between death due to suicide/non‐accidental injury and: sex (males > females, *p* < 0.001); race (persons of White ethnicity > Black or other ethnicity, *p* < 0.001); relationship status (persons never married > other relationship status, *p* < 0.001); disease stage (distant disease > localized disease, *p* < 0.001); or those with one tumor compared to those with multiple tumors (*p* = 0.014).

## 4 DISCUSSION

Self‐harm and suicide are a significant and growing issue among young people in the United States with 6241 deaths by suicide recorded among individuals aged 15–24 years in 2017. This corresponds to the highest suicide rate identified in this age group since 2000.^17^ In this paper, we report a more than 34 times increased rate of death from suicide/self‐inflicted injury among the US AYA cancer population compared to the general population (SMR 34.1), with a comparatively elevated risk persisting for this demographic over at least 5 years following a cancer diagnosis. Higher rates of death by suicide have been reported for patients of all ages with a cancer diagnosis than for the general population.^6^, ^7^ Young age is frequently reported as a risk factor for higher rates of psychological distress and mental ill‐health among adults with cancer.^1^ In addition to psychosocial risk factors, it has been proposed that neurodevelopmental factors pertinent to adolescence may additionally predispose this age group to increased vulnerability for self‐harm and suicidal behavior.^9^ These factors include increased risk‐taking behavior and ongoing maturation of emotional skills necessary to navigate major stressful life events such as a malignancy diagnosis.^9^, ^18^ While the long‐term psychological sequelae for childhood cancer survivors have been reported, little information exists in the literature related to either the rates or predictors for death by suicide specifically among those diagnosed with cancer during the AYA period.^11^, ^19^

Elevated death by suicide risk in AYA with cancer has likewise been demonstrated in a number of European studies.^7^, ^10^, ^18^, ^20^ The overall SMR result of 34.1 from our study is similar to an SMR of 37.2 for persons aged ≤39 years in a study by Zaorsky et al., which likewise utilized SEER registry data, however considered patients with cancer across all age groups.^7^ In comparison, Anderson et al. report a much lower SMR in AYA aged 15–39 years (1.23).^2^ This is likely explained by methodological differences due to the exclusion of patients who were alive or had unknown causes of death from their reference population. In our study, the reference population included anyone who had not died due to suicide/self‐inflicted injury in the reference population whether alive or deceased due to other causes.

In general, risk of death from suicide/non‐accidental injury decreased over time following a cancer diagnosis, affirming findings from other groups.^7^, ^18^ This trend was identified across most socio‐demographic and tumor variables, with a few exceptions. For individuals diagnosed with cancer at a younger age (15–29 years), risk appeared to remain elevated in a sustained fashion over time, whereas the highest risk of death by suicide occurred in the first year following a cancer diagnosis for older individuals aged 30–39 years. This may be of relevance to specialist oncology health‐care providers involved in follow‐up and survivorship care of patients diagnosed with cancer during the AYA period, as well as primary care providers. For patients with selected tumor types (CNS and associated tumors, soft tissue sarcomas, and osseous/chondromatous tumors) the highest risk fell within the second to fifth years post‐cancer diagnosis. In the study by Zaorsky et al., involving cancer patients of all ages, it is interesting to note by contrast that patients with Hodgkin’s lymphoma and prostate cancer demonstrated sustained elevation of risk, and patients with testicular cancer demonstrated increasing risk over time.^7^ A trend toward increased suicidal risk over time in the testicular cancer group is particularly intriguing, given in our study, risk decreased over time for AYA patients with germ cell and trophoblastic neoplasms.

The results of this study have important implications for the care of individuals who currently or previously have experienced a cancer diagnosis during the AYA years. While it may be noted that risk of death by suicide/self‐inflicted injury among this population generally decreased over time, substantial risk, beyond that of the general population, persisted for 5 years or more following diagnosis with cancer. It is noteworthy that at this juncture, for some individuals, the intensity of oncology‐specific follow‐up may have decreased, such as patients with early stage cancers who may be approaching completion of cancer surveillance schedules. Various factors complicate post‐treatment and survivorship care for this population in addition to standard survivorship concerns. These include a possibility of needing to transition to adult health facilities for some individuals, if diagnosed with cancer within the teenage years and initially managed by a pediatric oncology service; difficulties in navigating re‐integration into educational and vocational settings following disruptions due to treatment, and concerns for some regarding long‐term or ongoing medical complications of treatment such as cardiotoxicity or reduced fertility.^19^, ^21^ As such, effective survivorship programs will require tailoring to the specific needs of an AYA cancer population, of which routine surveillance for psychological concerns is clearly an imperative on the basis of this study’s findings, particularly within the first year after a cancer diagnosis. At the same time, given the persistence of elevated risk over at least the first 5 years post a cancer diagnosis, such models will likely require the engagement and awareness of multiple health‐care providers, not just those in cancer care.^22^ This raises complexities given an individual primary care physician’s exposure to AYA cancer may be limited, and thus also highlights the importance of initiatives aimed to empower patients and families to prompt concerns.^19^

A limitation that deserves acknowledgment in our analysis was the inability to consider the influence of mental health variables given these data are not captured within the SEER dataset. Higher frequency of suicidal ideation has been reported among cancer patients compared to the general population.^23^ In the psychiatric literature, mental health variables (prior non‐fatal suicide attempts, psychiatric comorbidities, and substance abuse) are frequently cited as having an association with suicide risk.^23^ As such, it is likely that consideration of pre‐existing mental health comorbidities may be relevant in evaluating factors associated with death by suicide in AYA patients with cancer. Despite this caveat, we would suggest that the results of our study serve to provide additional information regarding non‐mental health risk factors which clinicians and policy‐makers may consider when evaluating preventive interventions to address the aforementioned significantly elevated risk of death by suicide in AYA with cancer.

In summary, a greater than 30 times increased risk of death by suicide/non‐accidental injury among the AYA cancer population was identified and risk remained elevated for more than 5 years following a diagnosis with cancer. Consequently, monitoring of suicide risk, developing suicide prevention strategies, and tailoring psychosocial interventions addressing the mental health in AYA cancer patients are required across both oncology and primary care settings. While AYA account for a small proportion of annual cancer cases, the complexity of this subgroup places them at a disproportionately high risk of premature death and negative long‐term health outcomes.

## ETHICAL APPROVAL STATEMENT

Ethics approval was not required for this study given data utilized was a publicly available registry (Surveillance, Epidemiology, and End Results program).

## CONFLICT OF INTEREST

Jeremy Lewin: Consulting/advisory: Bayer. The rest of the authors have no disclosures or conflict of interest.

## Supporting information

Data S1

## Data Availability

The data that support the findings of this study are available in the Surveillance, Epidemiology, and End Results Program (SEER). These data were derived from the following resources available in the public domain: Surveillance Research Program, National Cancer Institute SEER*Stat software (seer.cancer.gov/seerstat) version 8.3.6.1
