# Supplementary material for: Risk factors associated with suicide in adolescents and young adults (AYA) with cancer
Source: Cancer Med. 2021 Sep 29;10(20):7339–46. doi: 10.1002/cam4.4246 (PMC8525084; doi:10.1002/cam4.4246)
Supplement: Supplementary file 1 — Data S1 [file CAM4-10-7339-s001.docx]

## **Supplementary Information and Data**

**Cross-sectional and longitudinal SMR analysis interpretation**

In our study it is pertinent to note also that high SMRs both in cross-sectional (Table 1, main manuscript) as well as longitudinal analysis (Figure 2, main manuscript), were noted in the more recent cohorts of patients (diagnosed with cancer during 2005-2016). This is likely driven by less person-years having been accrued compared to earlier diagnosis cohorts given the actual number of deaths during this period is fairly similar to earlier categories.

**Supplementary Data for Figure 2 Main Manuscript: Incidence of death by suicide/self-inflicted injury in AYA with cancer by demographic and clinical characteristics considered longitudinally from the date of cancer diagnosis**

|  |  |  |  |  |  |  |  |  | **Time since diagnosis** | |  |  |  |  |  |  |
| --- | --- | --- | --- | --- | --- | --- | --- | --- | --- | --- | --- | --- | --- | --- | --- | --- |
|  |  |  |  | **0 to 11 months** | |  |  |  | **12 to 59 months** | |  |  |  | **60 months and longer** | | |
|  |  | Suicides | SMR | (95% CI) | Person-years accrued |  | Suicides | SMR | (95% CI) | Person-years accrued |  | Suicides | SMR |  | (95% CI) | Person-years accrued |
|  | **All AYA patients with cancer** | 54 | 72.79 | (54.68, 94.98) | 193872.43 |  | 160 | 51.51 | (43.84, 60.14) | 742722.4 |  | 388 | 28.08 |  | (25.35, 31.02) | 2261665.79 |
|  | **Sex** |  |  |  |  |  |  |  |  |  |  |  |  |  |  |  |
|  | Male | 37 | 59.33 | (41.77, 81.78) | 78346.51 |  | 133 | 52.49 | (43.95, 62.21) | 285976.78 |  | 259 | 24.62 |  | (21.71, 27.81) | 856238.75 |
|  | Female | 17 | 143.79 | (83.76, 230.22) | 115525.92 |  | 27 | 47.17 | (31.09, 68.63) | 456745.62 |  | 129 | 39.12 |  | (32.66, 46.48) | 1405427.04 |
|  | **Race** |  |  |  |  |  |  |  |  |  |  |  |  |  |  |  |
|  | White | 49 | 72.75 | (53.82, 96.18) | 158796.2 |  | 142 | 49.67 | (41.83, 58.54) | 618378.87 |  | 370 | 28.96 |  | (26.09, 32.07) | 1951669.66 |
|  | Black | 1 | 17.89 | (0.45, 99.66) | 19133.14 |  | 11 | 53.54 | (26.72, 95.79) | 66161.61 |  | 9 | 10.06 |  | (4.60, 19.10) | 167726.83 |
|  | Other | 4 | 322.56 | (87.89, 825.89) | 15943.09 |  | 7 | 167.96 | (67.53, 346.07) | 58181.93 |  | 9 | 60.37 |  | (27.61, 114.61) | 142269.31 |
|  | **Relationship status** |  |  |  |  |  |  |  |  |  |  |  |  |  |  |  |
|  | Single (never married) | 34 | 107.34 | (74.33, 149.99) | 73573.91 |  | 87 | 78.39 | (62.79, 96.69) | 267496.76 |  | 142 | 37.69 |  | (31.75, 44.43) | 738893.93 |
|  | Married | 10 | 31.89 | (15.29, 58.66) | 94461.38 |  | 55 | 37.53 | (28.27, 48.85) | 374681.42 |  | 175 | 21.81 |  | (18.70, 25.29) | 1221412.68 |
|  | Separated/Divorced/Widowed/Unmarried | 4 | 74.21 | (20.22, 189.99) | 12851.47 |  | 8 | 29.62 | (12.79, 58.37) | 48476.87 |  | 38 | 30.58 |  | (21.64, 41.98) | 158307.17 |
|  | Unknown | 6 | 104.06 | (38.19, 226.50) | 12985.67 |  | 10 | 38.32 | (18.38, 70.48) | 52067.36 |  | 33 | 42.05 |  | (28.95, 59.06) | 143052.01 |
|  | **Age at diagnosis** |  |  |  |  |  |  |  |  |  |  |  |  |  |  |  |
|  | 15-19 | 2 | 64.32 | (7.79, 232.33) | 12274.71 |  | 4 | 27.37 | (7.46, 70.07) | 47278.16 |  | 29 | 49.92 |  | (33.43, 71.69) | 156368.31 |
|  | 20-24 | 4 | 52.53 | (14.31, 134.50) | 20561.48 |  | 19 | 63.28 | (38.10, 98.81) | 80600.57 |  | 55 | 47.11 |  | (35.49, 61.33) | 267270.95 |
|  | 25-29 | 8 | 68.97 | (29.78, 135.91) | 34453.88 |  | 34 | 71.73 | (49.67, 100.23) | 134555.29 |  | 67 | 30.22 |  | (23.42, 38.37) | 435302.78 |
|  | 30-34 | 17 | 84.11 | (48.99, 134.66) | 52110.05 |  | 50 | 63.82 | (47.37, 84.15) | 197773.22 |  | 103 | 27.42 |  | (22.38, 33.26) | 604271.8 |
|  | 35-39 | 23 | 72.67 | (46.07, 109.04) | 74472.3 |  | 53 | 37.79 | (28.31, 49.44) | 282515.18 |  | 134 | 21.98 |  | (18.42, 26.03) | 798451.96 |
|  | **Year of diagnosis** |  |  |  |  |  |  |  |  |  |  |  |  |  |  |  |
|  | 1975-1984 | 6 | 28.59 | (10.49, 62.24) | 35262.68 |  | 25 | 24.64 | (15.94, 36.37) | 140812.36 |  | 142 | 18.11 |  | (15.26, 21.35) | 802332.25 |
|  | 1985-1994 | 22 | 73.7 | (46.18, 111.58) | 48486.54 |  | 65 | 56.52 | (43.62, 72.04) | 189907.38 |  | 138 | 31.48 |  | (26.45, 37.19) | 808456.87 |
|  | 1995-2004 | 11 | 68.24 | (34.07, 122.10) | 49557.12 |  | 34 | 49.17 | (34.05, 68.70) | 209687.81 |  | 87 | 57.61 |  | (46.15, 71.07) | 530339.27 |
|  | 2005-2016 | 15 | 207.46 | (116.11, 342.18) | 60566.08 |  | 36 | 144.06 | (100.90, 199.44) | 202314.85 |  | 21 | 249.44 |  | (154.41, 381.29) | 120537.41 |
|  | **Number of primary tumours** |  |  |  |  |  |  |  |  |  |  |  |  |  |  |  |
|  | Single | 53 | 75.9 | (56.86, 99.28) | 172885.14 |  | 155 | 53.7 | (45.58, 62.85) | 648203.16 |  | 364 | 29.47 |  | (26.52, 32.66) | 1858746.26 |
|  | Multiple | 1 | 22.94 | (0.58, 127.79) | 20987.29 |  | 5 | 22.75 | (7.39, 53.08) | 94519.24 |  | 24 | 16.35 |  | (10.48, 24.33) | 402919.53 |
|  | Surgery |  |  |  |  |  |  |  |  |  |  |  |  |  |  |  |
|  | **Surgery performed** | 22 | 43.23 | (27.09, 65.45) | 148958.11 |  | 97 | 42.22 | (34.24, 51.51) | 602091.51 |  | 301 | 27.61 |  | (24.58, 30.91) | 1882695.71 |
|  | Not Performed | 30 | 133.45 | (90.04, 190.51) | 42197.66 |  | 63 | 82.26 | (63.21, 105.25) | 131409.64 |  | 82 | 29.18 |  | (23.21, 36.23) | 340529.85 |
|  | None/unknown | 2 | 244.77 | (29.64, 884.18) | 2716.65 |  | 0 | 0 | (0.00, 85.68) | 9221.25 |  | 5 | 47.5 |  | (15.42, 110.85) | 38440.23 |
|  | **Cancer site** |  |  |  |  |  |  |  |  |  |  |  |  |  |  |  |
|  | Leukemias | 5 | 265.51 | (86.21, 619.62) | 7329.18 |  | 5 | 77.51 | (25.17, 180.89) | 21798.93 |  | 8 | 41.6 |  | (17.96, 81.96) | 44910.1 |
|  | Lymphomas | 7 | 53.95 | (21.69, 111.17) | 23753.08 |  | 26 | 47.73 | (31.18, 69.94) | 94124.4 |  | 64 | 31.96 |  | (24.61, 40.81) | 289487.01 |
|  | CNS/Intracranial/Intraspinal Neoplasms | 2 | 55.68 | (6.74, 201.14) | 8438.98 |  | 10 | 78.19 | (37.49, 143.79) | 29329.69 |  | 17 | 64.11 |  | (37.35, 102.65) | 64090.27 |
|  | Osseous and Chondromatous Neoplasms | 0 | 0 | (0.00, 303.60) | 2707.55 |  | 4 | 77.23 | (21.04, 197.74) | 9400.79 |  | 8 | 19.91 |  | (8.60, 39.24) | 26527.45 |
|  | Soft Tissue Sarcomas | 10 | 113.95 | (54.64, 209.56) | 11922.73 |  | 33 | 144.32 | (99.35, 202.69) | 35069.19 |  | 19 | 40.82 |  | (24.57, 63.74) | 96420.53 |
|  | Germ Cell and Trophoblastic Neoplasms | 3 | 23.49 | (4.85, 68.66) | 17723.85 |  | 19 | 32.29 | (19.44, 50.42) | 75017.84 |  | 74 | 23.68 |  | (18.59, 29.73) | 264747.57 |
|  | Melanoma and Skin Carcinomas | 4 | 46.74 | (12.73, 119.66) | 23251.82 |  | 9 | 20.86 | (9.54, 39.60) | 99096.26 |  | 56 | 26.42 |  | (19.96, 34.31) | 334006.66 |
|  | Carcinomas | 23 | 96.7 | (61.30, 145.10) | 95660.91 |  | 52 | 49.97 | (37.32, 65.53) | 367520.49 |  | 138 | 26.69 |  | (22.42, 31.53) | 1111664.07 |
|  | Miscellaneous specified neoplasms, NOS | 0 | 0 | (0.00, 841.39) | 2345.56 |  | 1 | 45.56 | (1.15, 253.85) | 8647.07 |  | 3 | 54.18 |  | (11.17, 158.33) | 21198.45 |
|  | Unspecified Malignant Neoplasms | 0 | 0 | (0.00, 1879.04) | 737.93 |  | 1 | 163.17 | (4.13, 909.13) | 2713.74 |  | 1 | 49.74 |  | (1.26, 277.15) | 8608.55 |
|  | **Tumour grade** |  |  |  |  |  |  |  |  |  |  |  |  |  |  |  |
|  | Grade I | 1 | 20.3 | (0.51, 113.12) | 14065.71 |  | 9 | 39.67 | (18.14, 75.30) | 57827.47 |  | 31 | 21.83 |  | (14.83, 30.99) | 180592.89 |
|  | Grade II | 4 | 52.49 | (14.30, 134.40) | 22788.78 |  | 22 | 69.53 | (43.57, 105.26) | 87789.21 |  | 33 | 26.69 |  | (18.37, 37.48) | 224894.43 |
|  | Grade III | 7 | 141.74 | (56.99, 292.03) | 22323.42 |  | 8 | 36.72 | (15.85, 72.36) | 76872.94 |  | 29 | 24.11 |  | (16.15, 34.63) | 179220.42 |
|  | Grade IV | 2 | 99.02 | (11.99, 357.71) | 6135.99 |  | 7 | 107.37 | (43.17, 221.22) | 19127.05 |  | 7 | 28.46 |  | (11.44, 58.63) | 49072.44 |
|  | Other Cell (T-, B-, Null, N K) | 5 | 166.7 | (54.13, 389.02) | 8716.18 |  | 7 | 53.03 | (21.32, 109.27) | 29768.33 |  | 11 | 41.6 |  | (20.77, 74.43) | 52328.24 |
|  | Ungraded | 35 | 67.72 | (47.17, 94.18) | 119842.34 |  | 107 | 49.82 | (40.83, 60.20) | 471337.4 |  | 277 | 29.32 |  | (25.97, 32.98) | 1575557.38 |
|  | **Stage** |  |  |  |  |  |  |  |  |  |  |  |  |  |  |  |
|  | Localised | 15 | 48.37 | (27.07, 79.78) | 86816.69 |  | 46 | 30.93 | (22.64, 41.25) | 375603.16 |  | 201 | 27.9 |  | (24.17, 32.03) | 1269315.13 |
|  | Regional | 4 | 37.93 | (10.33, 97.12) | 38680.38 |  | 28 | 64.44 | (42.82, 93.13) | 147521.59 |  | 56 | 21.61 |  | (16.32, 28.06) | 402459.02 |
|  | Distant | 10 | 198.48 | (95.18, 365.01) | 19937.44 |  | 14 | 73.94 | (40.42, 124.05) | 54095.43 |  | 22 | 28.28 |  | (17.72, 42.82) | 116946.15 |
|  | Unknown/Unstaged | 25 | 90.61 | (58.64, 133.76) | 48437.92 |  | 72 | 72.37 | (56.63, 91.14) | 165502.22 |  | 109 | 33.6 |  | (27.59, 40.53) | 472945.5 |
